# Construction of millimeter-scale vascularized engineered myocardial tissue using a mixed gel

**DOI:** 10.1093/rb/rbad117

**Published:** 2023-12-29

**Authors:** Ming Ke, Wenhui Xu, Yansha Hao, Feiyang Zheng, Guanyuan Yang, Yonghong Fan, Fangfang Wang, Zhiqiang Nie, Chuhong Zhu

**Affiliations:** Department of Anatomy, Third Military Medical University, Chongqing 400038, China; Department of Anatomy, Third Military Medical University, Chongqing 400038, China; Department of Anatomy, Third Military Medical University, Chongqing 400038, China; Department of Anatomy, Third Military Medical University, Chongqing 400038, China; Department of Anatomy, Third Military Medical University, Chongqing 400038, China; Department of Anatomy, Third Military Medical University, Chongqing 400038, China; Department of Anatomy, Third Military Medical University, Chongqing 400038, China; Department of Anatomy, Third Military Medical University, Chongqing 400038, China; Department of Anatomy, Third Military Medical University, Chongqing 400038, China; State Key Laboratory of Trauma, Burn and Combined Injury, Chongqing 400038, China; Department of Plastic and Aesthetic Surgery, Southwest Hospital, Third Military Medical University, Chongqing 400038, China; Engineering Research Center of Tissue and Organ Regeneration and Manufacturing, Ministry of Education, Chongqing 400038, China

**Keywords:** engineered myocardium, vascularized, hiPSC-CM, HUVEC, fibrin

## Abstract

Engineering myocardium has shown great clinal potential for repairing permanent myocardial injury. However, the lack of perfusing blood vessels and difficulties in preparing a thick-engineered myocardium result in its limited clinical use. We prepared a mixed gel containing fibrin (5 mg/ml) and collagen I (0.2 mg/ml) and verified that human umbilical vein endothelial cells (HUVECs) and human-induced pluripotent stem cell-derived cardiomyocytes (hiPSC-CMs) could form microvascular lumens and myocardial cell clusters by harnessing the low-hardness and hyperelastic characteristics of fibrin. hiPSC-CMs and HUVECs in the mixed gel formed self-organized cell clusters, which were then cultured in different media using a three-phase approach. The successfully constructed vascularized engineered myocardial tissue had a spherical structure and final diameter of 1–2 mm. The tissue exhibited autonomous beats that occurred at a frequency similar to a normal human heart rate. The internal microvascular lumen could be maintained for 6 weeks and showed good results during preliminary surface re-vascularization *in vitro* and vascular remodeling *in vivo*. In summary, we propose a simple method for constructing vascularized engineered myocardial tissue, through phased cultivation that does not rely on high-end manufacturing equipment and cutting-edge preparation techniques. The constructed tissue has potential value for clinical use after preliminary evaluation.

## Graphical abstract

**Figure:**
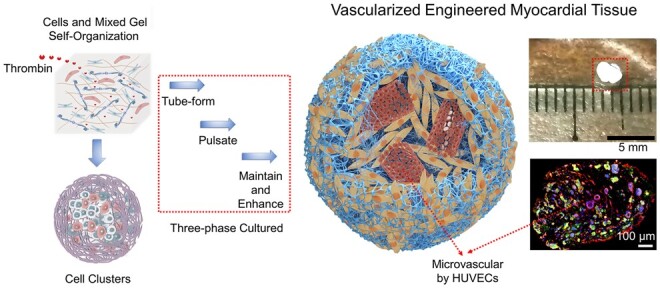

## Introduction

According to a World Health Statistics 2022 report, cardiovascular diseases are among the top three causes of death worldwide among chronic non-communicable diseases. In addition, permanent myocardial damage caused by myocardial infarction can lead to heart failure, which is life-threatening. At present, myocardial repair and heart transplantation are the main clinical therapeutics; however, they are limited by a shortage of donor organs and tissues. Therefore, over the past two decades, researchers have aimed to solve these issues by developing functionally engineered cardiac tissues *in vitro* [1].

Cardiac cells possess an innate capacity to re-establish a complex, 3D cardiac organization *in vitro* [2]. With advances in engineering technology and new materials, more complex and mature engineered myocardial tissues have been developed, representative types, myocardial microspheres, myocardial patches, myocardial strips in the form of rings, ribbons or cables and engineered human hearts [3–6]. Myocardial microspheres and thin myocardial patches have shown good effects for the treatment and improvement of cardiac functions in experimental animals [7, 8]. However, microspheres and thin-engineered myocardial tissue cannot repair a deeply damaged myocardium or replace partially thick cardiac tissue [9, 10]. Therefore, it is necessary to develop thick-engineered myocardial tissue (with a thickness exceeding 1 mm). However, the size limit of engineered tissue to maintain growth through free diffusion is 200 μm [11]. Therefore, a functional thick-engineered myocardium requires vascularization [12].

A traditional approach to constructing a thick-engineered myocardium using matrix materials and seed cells requires two steps. First, the seed cells and matrix materials need to be allowed to form spherical or sheet myocardial tissue. Then, this is reassembled into thick tissue through overlapping [13], curling myocardial patches layer-by-layer [14] or patterning [15]. The reassembly process can also add vascular structures as needed [16]. However, there are still shortcomings. For example, owing to the lack of mechanical properties, such as rigidity and elasticity, of the microvessels formed through the co-culture of endothelial cells or implanted vascular segments, these lumens are prone to atrophy and collapse, although their tubular structures in the engineered myocardium can be maintained for a long time [17]. More advanced technology comprises pre-vascularizing the engineered myocardium by embedding polymer materials. This method can also be used to generate a hierarchical vasculature in the engineered myocardial tissue [18, 19]. However, this construction method and the use of polymer materials with too much rigidity and insufficient elasticity can interrupt the natural connections between myocardial cells, ultimately affecting their contractility and conductivity [20, 21]. Moreover, the construction and cultivation of this pre-vascularized engineered myocardium require the use of advanced three-dimensional (3D) printing technology [22–24] or complex microphysiological systems (organ-on-a-chip) [25].

Fibrin is a natural biomaterial, it has been widely used in tissue engineering because of its biocompatibility, degradability, and extraordinary ductility and elasticity [26–28]. The use of human-induced pluripotent stem cell-derived cardiomyocytes (hiPSC-CMs) can fundamentally avoid cell exclusion reactions and ethical issues in the field of regenerative medicine [29]. As such, they have been widely used for the construction of engineered myocardial tissues [30–32] and the regenerative repair of injured myocardial tissue [33].

**Figure 1.**
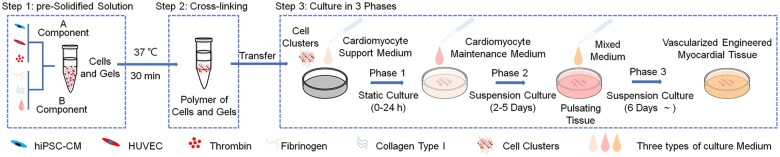
Steps for constructing vascularized engineered myocardial tissue.

Herein, we propose a simple method for constructing vascularized engineered myocardial tissue with a millimeter-scale spherical structure using hiPSC-CMs and human umbilical vein endothelial cells (HUVECs) that undergo self-organization in a mixed gel with fibrin. The main feature of this method is the phased cultivation of the engineered myocardial tissue (Figure 1); the use of fibrin (5 mg/ml) allowed us to harness its characteristics of low hardness under low shear forces, and ductility and elasticity under high shear forces (for more information, see Supplementary Information File: Section S1 and Section S2). During the static phase of cell self-organization, fibrin exhibited low hardness and HUVECs within the cell clusters formed rich lumen-like structures. However, during the dynamic contraction phase of hiPSC-CMs pulsation, fibrin ductility and elasticity allowed cell clusters to achieve spontaneous pulsation. Moreover, during the dynamic phase comprising the sustained spontaneous pulsation of cell clusters, lumen-like structures in the cell clusters expanded to form 50–200 μm microvessels that did not easily collapse. Ultimately, an engineered myocardial tissue with autonomous pulsation and lumen-like structures was obtained. We also achieved the *in vitro* surface re-vascularization and *in vivo* vascular remodeling of the vascularized engineered myocardial tissues. Our approach and the vascularized engineered myocardial tissue that was constructed will make it possible for multiple tissues to assemble and re-vascularize to form a larger and thicker-engineered myocardium in the future.

## Materials and methods

### Preparation of cell suspensions

hiPSC-CMs were obtained from commercial products (JK Med, China) and could be observed to be spontaneously beating under a microscope (Supplementary Information File: Section S3). After washing in 1× dulbecco’s phosphate buffered saline (DPBS, Gibco, USA) twice for 5 min, hiPSC-CMs were digested with EDTA Solution (Stemcell Technologies, Canada) for 10 min, followed by the addition of cardiomyocyte support medium (Stemcell) to terminate digestion, centrifugation to remove the supernatant, and dilution of the hiPSC-CM suspensions with cardiomyocyte support medium.

The primary HUVECs were obtained from commercial products (JK Med), and passages 4–7 were used for the experiments (Supplementary Information File: Section S3). HUVECs were cultured in Endothelial Cell growth Medium-2 (EGM-2, CC-3162, Lonza, Switzerland) supplemented with 1% penicillin–streptomycin solution (Hyclone, China). HUVECs were grown to 90% confluence before use in cell culture flasks. After washing in 1× DPBS twice for 5 min, the HUVECs were removed from the cell culture flask using 0.25% trypsin solution (Saimike, China). Then, dulbecco’s modified eagle medium: F-12 (DMEM/F12, Hyclone) with 10% fetal bovine serum (FBS, Hyclone) was added to terminate digestion, the samples were centrifuged to remove the supernatant, and HUVEC suspensions were diluted with EGM-2.

### Preparation of fibrinogen solution and thrombin solution

Approximately 10 ml of 0.9% NaCl solution (Saline solution) and 500 μg of bovine fibrinogen powder (500 mg, Solarbio, China) were slowly dissolved in a 50 mg/ml of bovine fibrinogen solution. The solutions were sterile-filtered using 0.22 μm pore filters. The entire process was conducted under sterile conditions at 37°C. Furthermore, 1000 U of thrombin powder (1000 U, Solarbio) was dissolved in 10 ml 0.9% NaCl solution to prepare a 100 U/ml thrombin solution at 25°C. The prepared solution was packaged and frozen for storage.

### Verification of 3D cell growth in a mixed gel with fibrin

To observe the 3D growth of hiPSC-CMs, circular climbing sheets were placed in 24-well cell culture plates. Approximately 470 μl hiPSC-CM suspensions (2 × 10^7^ cells/ml), 30 μl thrombin solution, 100 μl bovine fibrinogen solution, 20 μl collagen І, high concentration, rat tail (collagen І; 10.8 mg/ml, 354249, Corning, USA) and 380 μl cardiomyocyte support medium were thoroughly mixed on ice to form a pre-polymerized solution. The component concentrations of the pre-polymerization solution were as follows: hiPSC-CMs, 1 × 10^7^ cells/ml; fibrin gel, 5 mg/ml. Furthermore, 330 μl pre-polymerization solution was added to each well for static culture at 37°C with 5% CO_2_ for 30 min. After the pre-polymerization solution had solidified, 1 ml cardiomyocyte support medium was added to each well for static culture at 37°C with 5% CO_2_ for 24 h. Next, the cardiomyocyte support medium was removed, and fresh cardiomyocyte maintenance medium (Stemcell) was used to replace the medium every other day (1 ml/well). The growth process and spontaneous beating behavior of hiPSC-CMs in gels were observed through microscopy from Day 0 to 3.

To observe the 3D growth of HUVECs, circular climbing sheets were placed in 24-well cell culture plates. Then, 470 μl HUVEC suspensions (1 × 10^7^ cells/ml), 30 μl thrombin solution, 100 μl bovine fibrinogen solution, 20 μl collagen I and 380 μl EGM-2 were thoroughly mixed on ice to form a pre-polymerized solution. The component concentrations of the pre-polymerization solution were as follows: HUVECs, 5 × 10^6^ cells/ml; fibrin gel, 5 mg/ml. Furthermore, 330 μl pre-polymerization solution was added into each well for static culture at 37°C with 5% CO_2_ for 30 min. After the pre-polymerization solution had solidified, 1 ml EGM-2 was added to each well for static culture. Fresh EGM-2 was used to replace the medium every other day (1 ml/well). The growth process and formation of the ring- and lumen-like structures of HUVECs in the gels were observed through microscopy from Day 0 to Day 7.

### Construction and culture of vascularized engineered myocardial tissue

For cell self-organization in the mixed gel with fibrin, ∼250 μl hiPSC-CM suspensions (4 × 10^7^ cells/ml) and 250 μl HUVEC suspensions (2 × 10^7^ cells/ml), 30 μl thrombin solution, 100 μl bovine fibrinogen solution, 20 μl collagen I and 350 μl EGM-2, were mixed thoroughly on ice to form a pre-polymerized solution. The cell densities of the pre-polymerized solutions were as follows: hiPSC-CMs, 1 × 10^7^ cells/ml; HUVECs, 5 × 10^6^ cells/ml. Then, 400 μl pre-polymerized solution was added to an Eppendorf tube (EP, 1.5 ml). After solidification of the pre-polymerized solution, the polymer (engineered myocardial tissue) was placed into a well of a 24-well cell culture plates.

Three-phase cultivation was performed as follows: (i) 2 ml cardiomyocyte support medium was added to each well, and the engineered myocardial tissues were subjected static cultured for 24 h. (ii) The cardiomyocyte support medium was removed, 2 ml cardiomyocyte maintenance medium was added to each well, and the engineered myocardial tissues were suspension cultured for 2–5 days. Fresh cardiomyocyte maintenance medium was replaced every other day. (iii) On the sixth day, the cardiomyocyte maintenance medium was removed, 2 ml mixed medium (cardiomyocyte maintenance medium and EGM-2, v:v = 2:1) was added to each well, and the engineered myocardium tissues were suspension cultured. Fresh mixed medium was replaced every other day. All cultivation stages were performed at 37°C with 5% CO_2_ (Figure 1). The engineered myocardial tissue growth process and spontaneous beating behavior were observed via microscopy from Day 0 to Day 42.

### Pressure testing of vascularized engineered myocardial tissue

The elastic strain of the tissues, after static culture for 7 days, was measured using a BOSE ElectroForce (TA Instruments, USA). Three tissues were sequentially placed on the test bench of the equipment for the compression test, and each tested sample was compressed once. The testing parameters were as follows: compression distance, 0–0.35 mm; sample frequency, 200 points/s. The stress of each sample as a function of the compression distance was recorded using the manufacturer’s software.

### Cytological analysis of vascularized engineered myocardial tissue

The tissues of the cultured experimental and control groups were fixed and then frozen or paraffin-embedded and sliced. The immunofluorescence (IF) staining results based on an hiPSC-CM-specific antibody (cardiac Troponin T, cTnT) and HUVEC-specific antibody (VE-Cadherin, CD31) were used to evaluate vascularization of the engineered myocardial tissue. The number, area, and distribution of lumens formed by tightly arranged nuclei were statistically analyzed.

### Evaluation of re-vascularization *in vitro*

The micro-chip evaluation model was constructed in three steps. (i) Five vascularized engineered myocardial tissues (experimental group) and five non-vascularized (HUVEC-free) engineered myocardial tissues (control group) were cultured for 5 days. The size of the tissues was ∼500 μm. All experimental steps, reagents, and consumables in the control group were identical to those in the experimental group (Supplementary Information File: Section S4). (ii) Next, 1 ml of pre-polymerized solution containing HUVECs was prepared, with components as follows: HUVEC suspensions, 1 × 10^7^ cells/ml; thrombin, 3 U/ml; bovine fibrinogen, 5 mg/ml; collagen I, 0.2 mg/ml. The pre-polymerized solution was injected into the single-channel Polydimethylsiloxane (PDMS, Sylgard 184, Dow Corning, USA) micro-chips (Supplementary Information File: Section S5), filling the chamber. Then, five vascularized engineered myocardial tissues and five non-vascularized engineered myocardial tissues were respectively injected into the entrances of the two micro-chips. The pre-polymerized solution was continuously injected into the micro-chips, and the injection was stopped when the pre-polymerized solution pushed the tissues to the center of the chamber. The excess pre-polymerized solution at the inlet and outlet of the micro-chip was removed. (iii) The chips were placed in a disposable culture dish at 37°C with 5% CO_2_ for 30 min. After the pre-polymerized solution had solidified, the mixed medium was added to the culture dish to cover the micro-chips completely. The fresh mixed medium was replaced every 3 days. For cytological analysis, the growth process of HUVECs wrapped in the tissues was observed using microscopy from Day 0 to 5. At predetermined time points, the number and length of lumen-like structures formed by HUVECs on the surface of tissues, as well as the wrapping thickness, were evaluated.

### Evaluation of vascular remodeling ability *in vivo*

The animal experiments were carried out in accordance with the Laboratory Animal Administration Rules of China and approved by the Ethics Committee of the Third Military Medical University (AMUWEC20201449).

During preparation of the gel membrane with the tissue, to prevent rapid degradation of the tissue *in vivo*, the vascularized engineered myocardial tissue was wrapped in a mixed gel to create a gel membrane. First, 100 μl bovine fibrinogen solution, 30 μl thrombin solution, and 870 μl mixed medium were thoroughly mixed on ice to form a pre-polymerized solution. Then, 500 μl pre-polymerized solution was placed in a culture dish (35 mm). Then, the tissue, ∼1.5 mm, was placed in the pre-polymerized solution, and shaken slowly to embed it into the pre-polymerized solution at 37°C in 5% CO_2_ for 30 min. After the pre-polymerized solution solidified, the mixed gel formed a membrane with the tissue at the bottom of the culture dish. Next, the mixed medium was added to the culture dish, and static culture was continued for 1 day.

For subcutaneous implantation, six 20-day-old SD rats (male or female) were used. From the day before the implantation experiment, immunosuppression in each rat was induced via an intraperitoneal injection of cyclosporine solution. Rats were anesthetized using an intravenous injection of anesthetics. The hair on the back of the rat skin was removed, and the skin was exposed. After wiping the exposed skin with iodophor, an opening ∼1 cm in length was cut using surgical scissors. Surgical forceps were used to implant the gel membrane with tissue into the skin, and a suture was used to suture the wound. Erythromycin ointment (Pythonbio, China) was applied to the wound to prevent infection. Animals were then placed in cages for routine husbandry and administered daily intraperitoneal injections of a cyclosporine solution.

For histological analysis, the rats were anesthetized after 7 days, the skin was clipped from the extended suture wounds, and the skin with implants was clipped into 4% paraformaldehyde (PFA; Biosharp, China). The status of blood vessels growing into tissue and the activity of hiPSC-CMs were evaluated by IF and hematoxylin and eosin (H&E) staining.

### Fixing, sectioning, IF staining and H&E staining

*Fixing*: (i) The engineered myocardial tissues and animal tissues were fixed in 4% PFA for 1 h at room temperature. (ii) Cultures on climbing pieces and inside the micro-chips were fixed with 4% PFA for 15 min at room temperature.

*Sectioning*: Fixed tissues were subjected to gradient dehydration. They were then flash-frozen in Tissue-Tek OCT compound (Sakura, Japan) or subjected to paraffin embedding (technical support provided by Wuhan Servicebio Technology Co., Ltd, China), with serial sections (8–10 μm thickness). Each tissue sample was sliced horizontally and vertically.

*IF staining*: The laboratory procedure was as follows: The sections were washed with 1× phosphate buffer saline (PBS, Hyclone) (three times for 5 min), permeabilized with 0.1% Triton X-100 (Beyotime, China) for 20 min at 25°C, then washed again with 1× PBS (three times for 5 min). They were then blocked with a 3% bovine albumin (BioFroxx, China) solution for 1 h before primary antibody dilutions were added. The primary antibodies used for hiPSC-CMs were anti-cTnT (1:400, MA5-12960, Invitrogen), anti-α-SA (1:400, ab10135, Abcam, UK); the primary antibodies used for HUVECs were anti-Von Willebrand factor (vWF, 1:400, ab6994, Abcam), anti-Human CD31 (1:400, BBA7, R&D, USA) and anti-CD144 (1:400, 2500s, CST, USA). The primary antibody was aspirated after overnight incubation at 4°C, and the stained slides were then washed with 1× PBS (three times at 5 min). Secondary antibodies (1:500, Invitrogen, USA) corresponding to the primary antibodies and TRITC phalloidin (1:500, CA1610, Solarbio) were diluted in 1× PBS. Stained samples were incubated with the corresponding secondary antibody dilutions or sequentially with the corresponding secondary antibody dilutions and TRITC-phalloidin dilutions, as required for the experiment, at room temperature for 2 h. The secondary antibody was then aspirated, and 4′,6-diamidino-2-phenylindole (DAPI, 10 μg/ml, Biosharp) was added for 3 min. The samples were then washed with 1× PBS (three times for 5 min) before adding an antifade solution to seal them.

*H&E staining*: Samples were sequentially stained with hematoxylin and eosin, followed by dehydration, hyalinizing and neutral gum-mounting (technical support provided by Wuhan Servicebio Technology Co., Ltd).

### Imaging

Random images and video recordings of the cultures were captured at the time of fixation using optical and confocal scanning microscopy (Olympus, Japan). The 3D reconstruction images were created with technical support provided by the Chongqing University Cancer Hospital (Leica, Germany). At least three images were captured for each sample. Video recordings were at least 3 min in length. All images and videos were calculated using Image J software (National Institutes of Health).

### Statistical analysis

Data were analyzed using GraphPad Prism 8.0.1 (GraphPad Software, USA) to calculate mean ± standard deviation. One-way analysis of variance followed by Tukey’s *post hoc* analysis and *t* tests were used to determine statistically significant differences. Statistical significance was set at follows: ns, *P* > 0.05; *0.01 < *P* ≤ 0.05; **0.001 < *P* ≤ 0.01; ***0.0001 < *P* ≤ 0.001; and ****P ≤ 0.0001.

## Results

### HUVECs grew as 3D cultures in a mixed gel with fibrin

**Figure 2.**
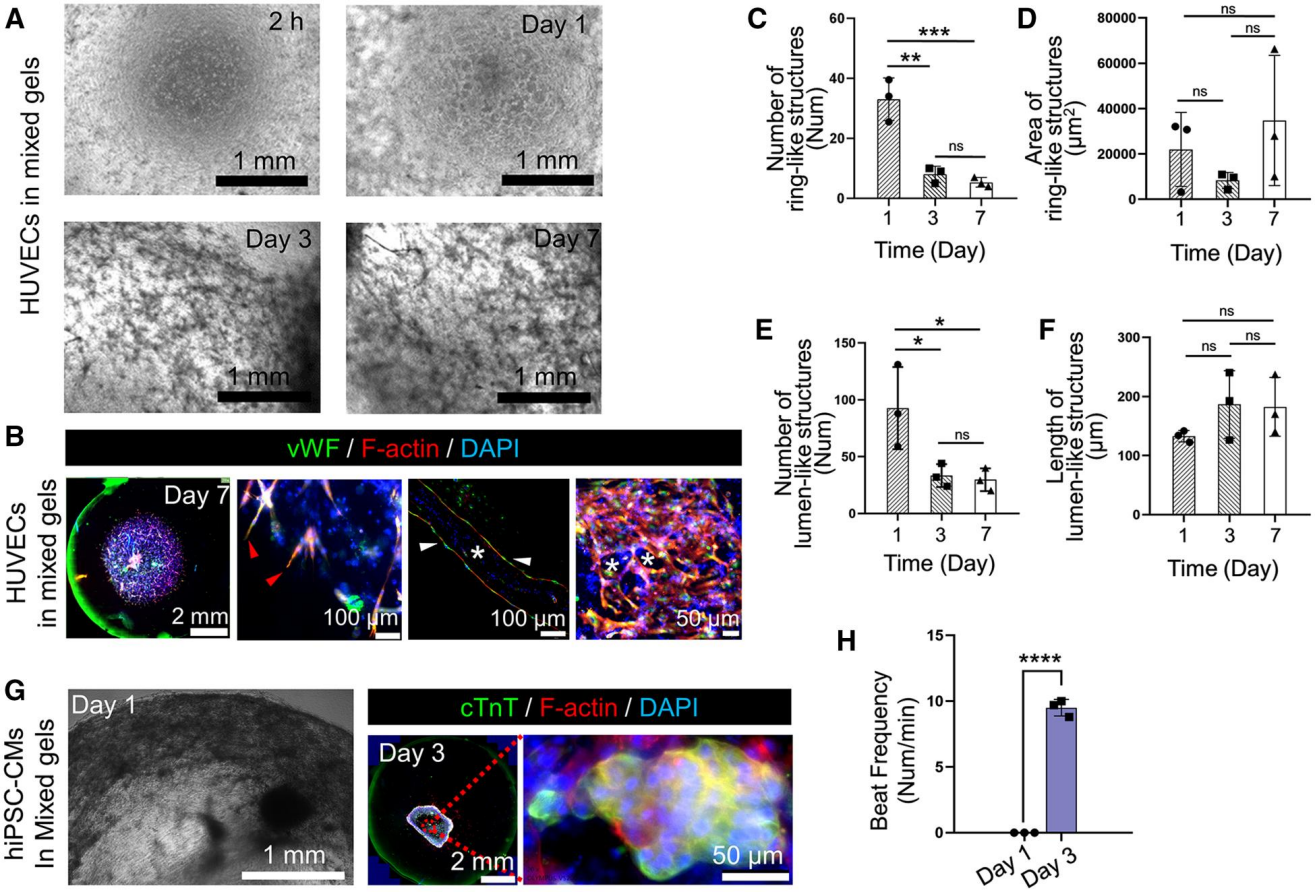
Human umbilical vein endothelial cells (HUVECs) and human-induced pluripotent stem cell-derived cardiomyocytes (hiPSC-CMs) both grew (3D) in a mixed gel with 5 mg/ml fibrin. (**A**) Microscopic images of the HUVEC growth process in the mixed gel. (**B**) Immunofluorescence (IF) image of HUVEC budding (triangles) and the formation lumen-like (triangles and asterisk) and ring-like (asterisks) structures in the mixed gel on the seventh day. (**C**–**F**) Columnar statistical plot of HUVECs forming ring- and lumen-like structures in the mixed gel from 1 to 7 days. (**G**) Microscopic image and IF staining of hiPSC-CMs showing 3D growth in the mixed gel on Days 1 and 3. (**H**) Columnar statistical plot of spontaneous hiPSC-CMs beating in the mixed gel on Days 1 and 3. (ns, P > 0.05; *0.01 < P ≤ 0.05; **0.001 < P ≤ 0.01; ***0.0001 < P ≤ 0.001; and ****P ≤ 0.0001)

3D images of the HUVEC growth process in the mixed gel, from 2 h to 7 days, were recorded using a microscope (Figure 2A). On the seventh day, IF staining was performed for the specific HUVEC markers vWF and F-actin, which showed budding, along with lumen- and ring-like structures (Figure 2B). The data showed that HUVECs were pulled to form ring- and lumen-like structures on the first day. From the third to the seventh day, the number of ring- and lumen-like structures formed by HUVECs decreased compared to those in the first day, but there was no significant differences in the average area of ring-like structures or average length of lumen-like structures (Figure 2C–F). These findings confirm that the mixed gel with 5 mg/ml fibrin is suitable as a 3D culture substrate for HUVEC growth and the formation of microvascular networks and can be used to construct vascularized tissue.

### hiPSC-CMs grew as 3D cultures in a mixed gel with fibrin

hiPSC-CMs were cultured in a mixed gel for 1–3 days, and the cultures exhibited apparent shrinkage and detached edges to the center (Figure 2G). IF staining was performed for the specific hiPSC-CMs markers cTnT and F-actin to characterize the 3D growth of hiPSC-CMs in the mixed gel. Spontaneous beating of the hiPSC-CMs within the mixed gel was observed on the third day, but there was no spontaneous beating on the first day (Figure 2H). This confirms that the mixed gel containing 5 mg/ml fibrin is suitable for constructing pulsatile-engineered myocardial tissue *in vitro*.

### Constructed millimeter-scale engineered myocardial tissue

**Figure 3.**
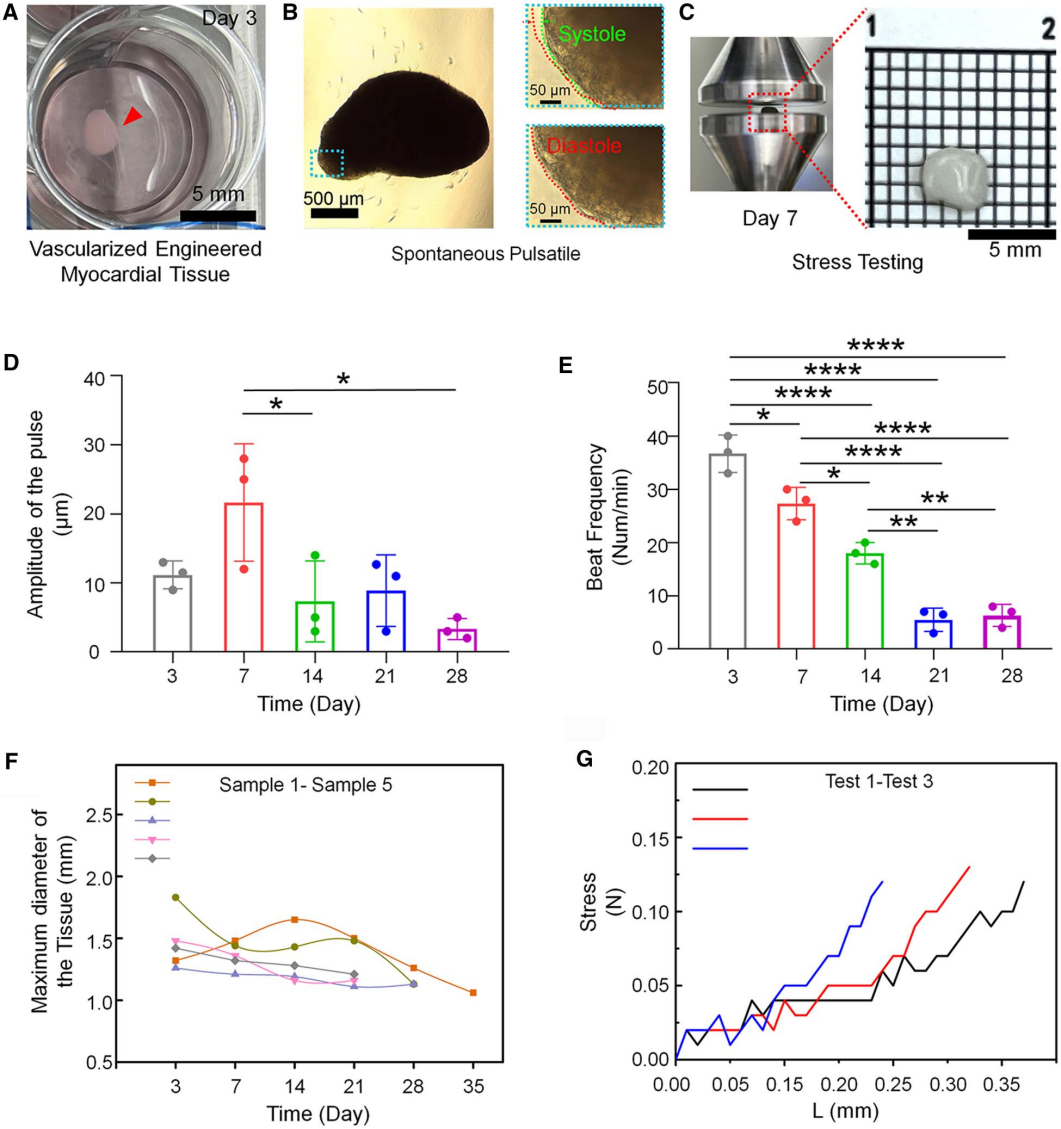
Appearance size dimension, spontaneous pulsation and mechanical characterization of millimeter-scale engineered myocardial tissues. (**A**) Millimeter-scale tissue during static culture. (**B**) Microscope images of the systolic and diastolic states of the tissue during spontaneous pulsation. (**C**) Tissue images during stress testing. (**D** and **E**) Changes in spontaneous pulsation amplitude and beat frequency of the tissues with static culture time. (**F**) Changes in millimeter-scale tissues' maximum diameter with the static culture time. (**G**) Changes in the pressure of the tissue with a changing compression distance. (*0.01 < P ≤ 0.05; **0.001 < P ≤ 0.01; and ****P ≤ 0.0001)

Multiple batches of engineered myocardial tissues were cultured and observed for more than 3 weeks. In the first cultivation phase (approximately starting on the first day), millimeter-scale cell clusters were formed in a well of the 24-well cell culture plate (Figure 3A). In the second cultivation phase (approximately starting on the third day), a spontaneous systole and diastole, similar to those of heart tissue, were observed under a microscope (Figure 3B and Supplementary Information: Movie S1). However, there were differences in the amplitude and beat frequency of different tissues, with the fastest beat frequency approaching a normal human heart rate, at ∼70 beats per minute (Supplementary Information: Movie S2).

The amplitude of the pulse reached its peak and then weakened after 7 days (Figure 3D), and the spontaneous beat frequency gradually slowed (Figure 3E and Supplementary Information: Movie S3). Moreover, with an increase in the culture time, the external structure and size of the tissues changed; the average shrinkage and collapse rate of the tissues was ∼30% (Figure 3F). This may be because of insufficient nutrient supply to the tissues and pulsating contractions of the tissues causing cell apoptosis.

To evaluate the hardness of the constructed tissue, compression experiments at a distance of 0–35 μm were conducted (Figure 3C). The tissues showed a mechanical response to pressure, as the compression distance increased. Therefore, the tissues had already achieved a certain degree of stiffness after 7 days of static cultivation (Figure 3G).

### Cytological evaluation of vascularized engineered myocardial tissue

**Figure 4.**
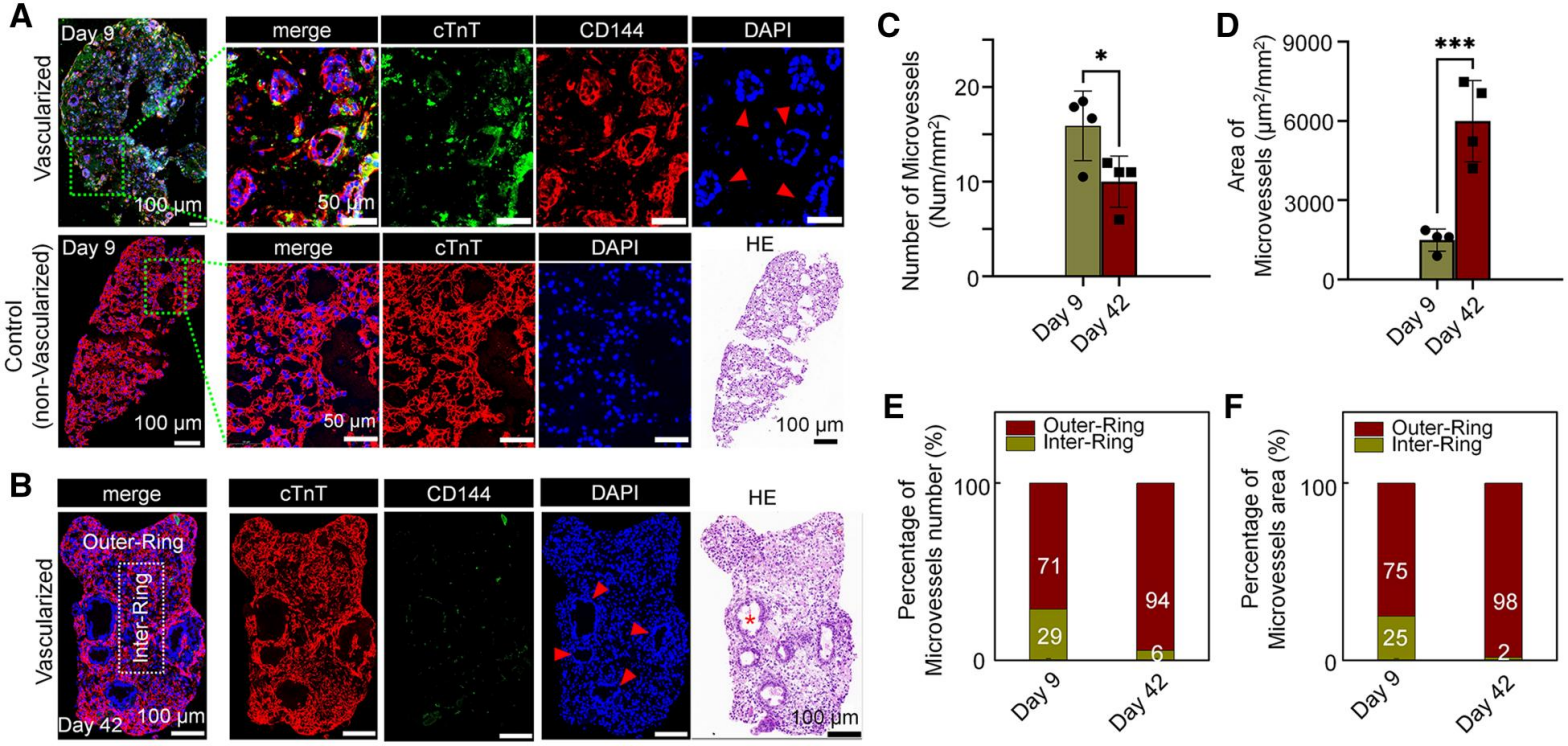
Microvascular structures formed in the engineered myocardial tissue. (**A**) Immunofluorescence (IF) and hematoxylin and eosin (H&E)-stained images of vascularized and human umbilical vein endothelial cell (HUVEC)-free engineered myocardial tissue sections on Day 9 of culture. Triangles mark lumen-like structures. (**B**) IF and H&E-stained images of vascularized engineered myocardial tissue sections on Day 42. The area within the dashed box is the inner-ring; the remaining areas are the outer-ring. Triangles and asterisk mark lumen-like structures. (**C** and **D**) Number and area of lumen-like structures in the tissues on the 9th and 42nd days. (**E**, **F**) Percentages of lumen-like structures in the inner- and outer-rings of the tissues on the 9th and 42nd days. (*0.01 < P ≤ 0.05; ***0.0001 < P ≤ 0.001)

IF staining results (CD144 of HUVECs and cTnT of hiPSC-CMs) of the engineering myocardium after 9 days of static cultivation showed that there were multiple lumen-like structures formed by HUVECs in the tissue, and the nuclei were clearly arranged in an orderly manner (red triangular pattern). In contrast, in the control group (HUVEC-free), the internal cells were loosely distributed, the boundary was discontinuous, and there were no densely arranged cavities formed by nuclei (Figure 4A). This indicates that the lumen-like structures in the engineered myocardial tissue were not voids or vacuoles.

To study the maintenance of the lumen-like structures formed in the engineered myocardial tissue, the internal structure after cultured for 42 days was identified. IF and H&E staining results showed that hiPSC-CMs still existed in the engineered myocardium, and multiple lumen-like structures were formed by tightly arranged nuclei in the tissue. However, expression of the specific marker CD144 did not occur around these nuclei, as shown in the triangular pattern (Figure 4B).

The number of lumen-like structures on the 42nd day was significantly lower than that on the ninth day (Figure 4C), and the area of lumen-like structures was significantly larger than that on the ninth day (Figure 4D). Meanwhile, differences occurred in the distribution of lumen-like structures within the same tissue. On the ninth day, more lumen-like structures were distributed in the outer-ring than in the inner-ring. However, on the 42nd day, lumen-like structures were almost all distributed in the outer-ring of the tissue (Figure 4E and F). This indicates that the lumen-like structures underwent changes during the culture process.

### Re-vascularization of the tissue surface *in vitro*

The re-vascularization ability of the vascularized engineered myocardial tissue surface was evaluated by investigating the ability of HUVECs in the mixed gel to form lumen-like structures on the tissue surface. The feasibility of this method was verified through pre-experiments (Supplementary Information File: Section S6).

**Figure 5.**
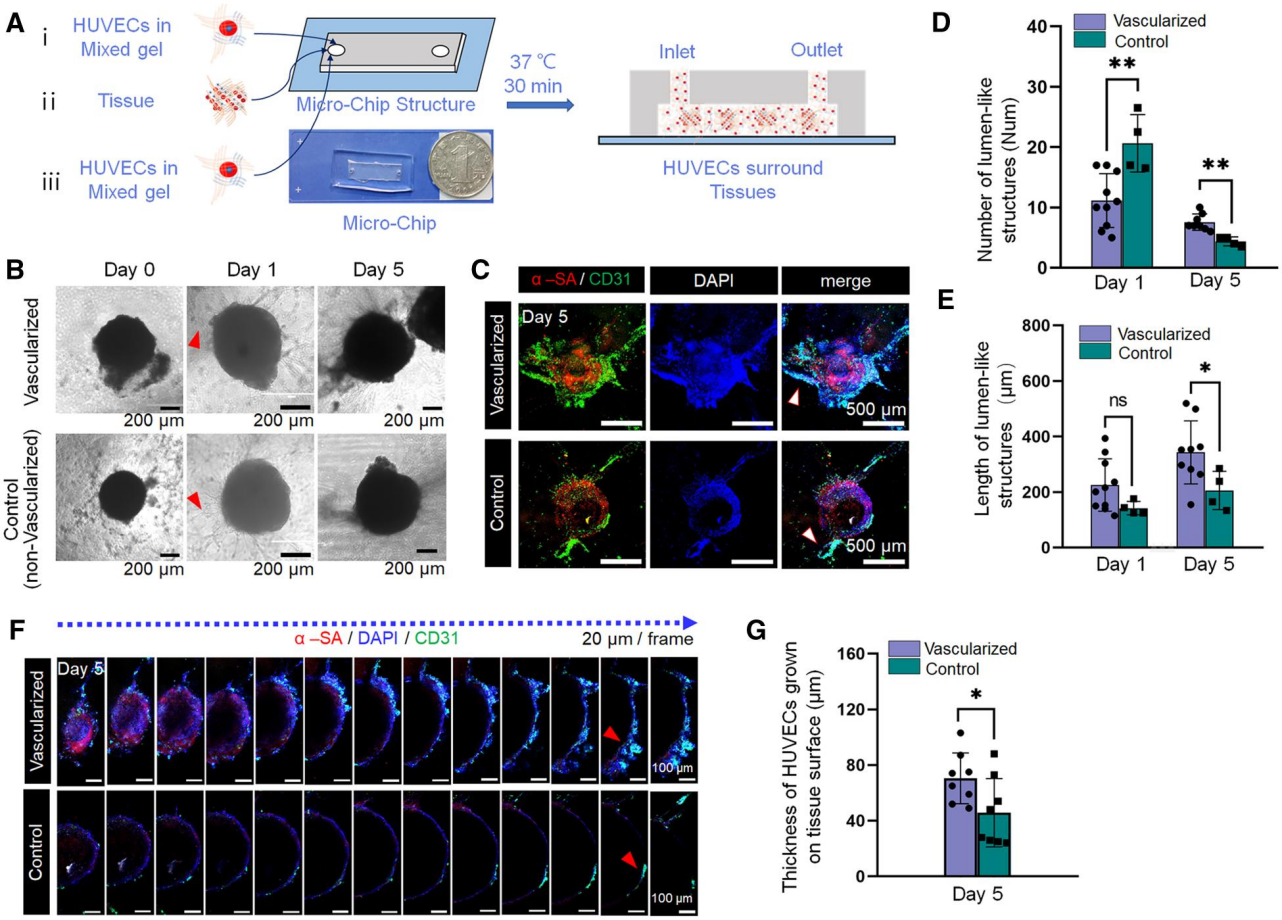
Re-vascularization on the surface of engineered myocardial tissue vascularized *in vitro*. (**A**) Schematic diagram of re-vascularization experimental model and micro-chip structure. (**B**) Microscopic images showing the process of human umbilical vein endothelial cell (HUVEC) re-vascularization on the surfaces of tissues. Triangular patterns mark lumen-like structures. (**C**) Three-dimensional (3D) images of immunofluorescence (IF) staining of re-vascularization mediated by HUVECs on the tissue surface on Day 5. Triangular patterns mark lumen-like structures. (**D** and **E**) Number and length of lumen-like structures on the tissue surface on the first and fifth days. (**F**) Cross-sectional images of IF staining of re-vascularization mediated by HUVECs on the tissue surface on the fifth day. (**G**) Thickness of HUVECs grown on tissue surface on the fifth day. (ns, P > 0.05; *0.01 < P ≤ 0.05; **0.001 < P ≤ 0.01)

The mixed gel containing HUVECs and multiple tissues was injected into a single channel micro-chip, step-by-step, for static culture (Figure 5A). HUVEC budding and lumen-like structures on the surfaces were observed in both the experimental (vascularized) and control groups (non-vascularized) on the first day (Figure 5B). However, the number of lumen-like structures in the vascularized group on the first day was significantly lower than that in the control group. On the fifth day, the number of lumen-like structures in the vascularized group was significantly higher than that in the control group (Figure 5D). On the first day, the lengths of lumen-like structures in the vascularized group were not significantly longer than those in the control group, but on the fifth day, the lengths of lumen-like structures in the vascularized group were significantly longer than those in the control group (Figure 5E).

Furthermore, we performed 3D reconstruction (Figure 5C) and scanning confocal microscopy (Figure 5F) based on IF staining. The results, based on the positive of the HUVEC-specific marker (CD31), showed that the thickness of HUVECs grown on the tissue surface in the vascularized group was significantly higher than that in the control group (Figure 5G). Moreover, HUVECs had a more compact growth trend on the tissue surface in the vascularized group.

### Vascular remodeling of vascularized engineered myocardial tissue *in vivo*

**Figure 6.**
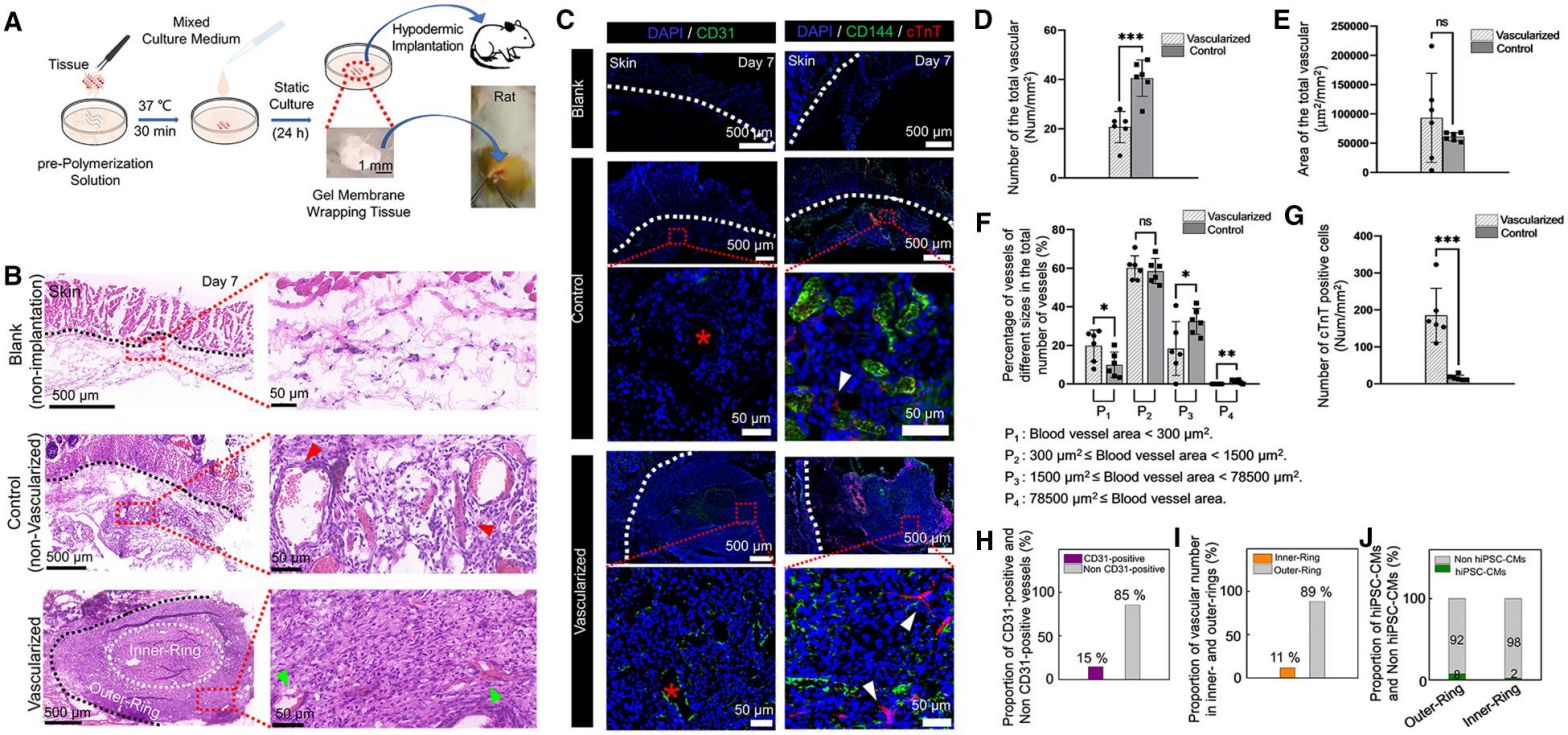
Vascular remodeling and the activity of human-induced pluripotent stem cell-derived cardiomyocytes (hiPSC-CMs) in vascularized engineered myocardial tissue *in vivo*. (**A**) Schematic diagram of a mixed gel-wrapped tissue and the subcutaneous implantation experiment. (**B**) Hematoxylin and eosin (H&E)-stained images of tissues obtained after subcutaneous implantation. The area within the white dashed box is the inner-ring; the remaining area is the outer-ring. (**C**) Immunofluorescence (IF) staining of implant. (**D** and **E**) The number and area of blood vessels in subcutaneous implant after 7 days. (**F**) The proportion of blood vessels with different diameters in the subcutaneous implant after 7 days. (**G**) cTnT-positive expression of hiPSC-CMs in the subcutaneous implant after 7 days. (**H**) The percentages of CD31-positive blood vessels in subcutaneous implant of the experimental group after 7 days. (**I**) The proportion of the number of blood vessels formed in the inner- and outer-rings in the subcutaneous implant of experimental group after 7 days. (**J**) The percentages of cTnT-positive hiPSC-CMs in the outer-and inner-rings compared with the number of cells in the implant of the experimental group after 7 days. (ns, P > 0.05; *0.01 < P ≤ 0.05; **0.001 < P ≤ 0.01; ***0.0001 < P ≤ 0.001)

The vascular remodeling ability of vascularized engineered myocardial tissue was studied *in vivo*. Briefly, the tissue was wrapped in mixed gel membrane, successfully implanted subcutaneously in the backs of rats, and grown for 7 days (Figure 6A). It was expected that the viability of the myocardium wrapped in fibrin would not be weakened [34]. We also verified that the tissue wrapped in a gel membrane exhibited spontaneous pulsation after 15 days *in vitro* culture (Supplementary Information: Movie S4).

The number and area of blood vessels in the tissue were evaluated by performing H&E staining of tissue slices at the implantation site (Figure 6B). In the blank group (non-implantation), the vascular network did not develop on the inner side of the skin layer in normal rats. In the control group (non-vascularized), loose tissue with vessels and blood cells (red triangles in Figure 6B) were observed at the subcutaneous implantation site. In the experimental group (vascularized), dense tissue with microvessels and a small number of branching blood vessels and blood cells (green triangles in Figure 6B) were observed at the subcutaneous implantation site. The IF staining results revealed CD31-positive endothelial cells in the experimental group, indicated by a red star in Figure 6C.

Although the number of blood vessels in the experimental group was significantly lower than that in the control group (Figure 6D), no significant difference in the blood vessel area was observed between the experimental and control groups (Figure 6E). However, the number of blood vessels with different diameters varied between the implants of experimental and control groups (Figure 6F): Specifically, the proportion of blood vessels within the microvascular size range (blood vessels with an area < 300 μm^2^) was significantly higher in the experimental group than that in the control group. Moreover, there was no significant difference in the proportion of blood vessels within the small vessel size range (blood vessels with an area of 300–1500 μm^2^) between the experimental and control groups. However, the proportion of blood vessels exceeding the small vessel size range (blood vessels with an area ≥ 1500 μm^2^) was significantly lower in the experimental group than that in the control group. We speculate that the large blood vessels with blood cells in the control group might have formed following inflammation in the rats. Notably, the blood vessels formed at the implantation site of the experimental group were similar to capillary structures in normal tissue.

The number of vessels with positive CD31 accounted for 15% of the total vessels count in the implant of the experimental group (Figure 6H), and more vessels formed in the outer-ring of the tissue than that in the inner-ring in the experimental group (Figure 6I). Our findings showed that the implantation sites of the vascularized and non-vascularized groups underwent vascular remodeling *in vivo*. These vessels were mainly formed by the rats themselves.

### hiPSC-CMs survived after implantation

To evaluate the activity of hiPSC-CMs, the specific marker of these cells, cTnT, was analyzed at the implantation site. IF staining results (Figure 6C) showed that positive expression of this marker was not observed outside the skin layer of the blank group. However, in both the control and experimental groups, cTnT-expressing hiPSC-CMs were observed at the implant sites (white triangles). The experimental group had significantly more cTnT-expressing hiPSC-CMs at the implant than the control group (Figure 6G). This indicates that vascularized engineered myocardium is more conducive to the survival of hiPC-CMs. In the experimental group, the cTnT-expressing hiPSC-CMs accounted for 8% of the total cell count in the outer-ring, but accounted for only 2% of the total cell count in the inner-ring (Figure 6J). This may be attributed to the fact that vascular remodeling was more limited in the inner-ring compared to outer-ring. So, the nutrient supply was compromised in the inner-ring, resulting in a decrease in the number of live hiPSC-CMs.

## Discussion

Heart injury, such as myocardial infarction, leads to cardiomyocyte loss, fibrotic tissue deposition and scar formation [35]. One way to repair the damaged myocardium is to paste an engineered myocardial tissue patch on the damaged part. However, the reparative effect of myocardial patches on the damaged myocardium in deeper areas is not satisfactory, and a thicker-engineered myocardium might be needed to replace the myocardium in deeper areas [36]. A thick-engineered myocardium requires blood vessels to provide nutrients for the internal cells; thus, it is also necessary to construct rigid blood vessels within the thick-engineered myocardium [37]. However, the mechanical properties of microvessels spontaneously formed by endothelial cells or implanted microvascular fragments are insufficient to resist myocardial contraction and collapse, ultimately limiting effective perfusion in the thick-engineered myocardium. Although this problem can be avoided by implanting blood vessels with high-strength scaffold materials, the materials could affect myocardial contraction [38]. In addition, building a thick-engineered myocardium with perfusable blood vessels typically requires advanced technology and high-end equipment.

The multicellular clusters generated through cellular self-organization have recently been found to have cytoarchitectural complexities similar to those of native tissues [39]. Accordingly, this traditional method of constructing an engineered myocardium using cell self-organization is beginning to receive renewed attention [8]. Therefore, we constructed vascularized engineered myocardial tissue through self-organization and cultured seed cells by harnessing the low hardness and elasticity of fibrin. The key to this method is achieving the self-organization and clustering of seed cells, spontaneous tube formation of HUVECs and spontaneous pulsation of hiPSC-CMs through three specific culture cycles, providing mechanical stimulation to promote the formation of larger lumens [40]. Compared with the method of assembling a small and thin-engineered myocardium into a larger-sized vascularized engineered myocardium, with this method, HUVECs can form rich lumens in the initial spherical structure with a diameter of several millimeters followed by the pulsating myocardium providing tensile stress stimulation to the lumens formed promote their expansion. This can avoid the implantation of vascular structures during the assembly process and reduce difficulties associated with preparation. The tissue constructed herein had spherical cell clusters with a maximum original diameter of 3–4 mm, but the tissue shrank with an increase in the static culture time (after 6 weeks, their shrinkage rate was ∼30%), Because the engineering myocardium will shrink without support [15]. The viscoelasticity of the 5 mg/ml fibrin used herein was ∼1 kPa, which is similar to the mechanical properties of myocardial patches without polymer scaffold materials reported in the literature [41], but their mechanical strength is still lower than those of myocardial patches constructed from other biological scaffold materials [42].

The tissues also achieved rhythmic pulsation with a maximum pulsation frequency close to 70 beats/min, and the spontaneous pulsation lasted for at least 4 weeks. Compared with the pulsatile ability reported in the literature, in the tissue that we constructed without stimulation, the duration of spontaneous pulsation was longer, but its pulsatile force and frequency were lower [15]. This could be related to the immature functions of hiPSC-CMs [43]. The reasons for this immaturity might originate from different aspects as follows: the hPSC state before induction was not naïve pluripotency, which is conducive to differentiation [44], the cells were not purified during the reprogramming stage of iPSCs, and the mature hiPSC-CMs were not screened [32].

Based on cytological identification, there were multiple lumen-like structures with diameters of tens of microns in the engineered myocardial tissue after 9 days of culture *in vitro*. After 6 weeks of culture, the lumen inside the tissue was still present and the lumen diameter reached more than 100 μm. Typically, the lumen-like structures formed by primary HUVECs in biological scaffold materials are difficult to maintain for more than 2 weeks without the addition of growth factors [45]. We speculate that the tensile stress generated by myocardial pulsation promotes the expansion and fusion of the lumen. After 6 weeks of culture, the cells around the lumen barely expressed the specific protein CD144 of HUVECs, although the nuclei around the lumen were arranged neatly and closely, unlike around the cavity formed after cell apoptosis. We speculate that HUVECs may be undergoing mesenchymal transition or be in the apoptotic cycle. Further research is underway to explore the potential mechanisms behind these experimental phenomena.

The vascular remodeling ability of an engineered myocardium is a vital reference index for clinical use. Using microfluidic technology, the ability of HUVECs to form lumen-like structures on the surface of vascularized engineered myocardial tissue was confirmed *in vitro*, but the lumen-like structures within the tissue could not be perfused externally. Therefore, we are also updating the construction method, attempting to generate a circular structure of myocardial tissue [46], and then use the mixed gel containing HUVECs to wrap the circular tissue. This tissue structure might be conducive to the formation of internal and external perfusing vessels [15]. Alternatively, the fluid shear force can be used to stimulate endothelial cells and promote the formation of perfusable lumens [47, 48].

Further animal experiments showed that some red blood cells flowed into the microvessels in the tissue 7 days after subcutaneous implantation of the engineered myocardium in rats. This indicated that the implanted tissue had preliminarily achieved vascular remodeling. Meanwhile, the survival rates of hiPSC-CMs in tissues with vascular remodeling were higher than those without vascular remodeling, which was also consistent with the literature [49, 50]. However, the number of blood vessels in the inner-ring was significantly lower than that in the outer-ring of the implant (the thickness of the outer-ring is ∼500 μm). We speculate the following as reasons: (i) The HUVECs in the inner-ring of the implant might have undergone apoptosis due to immune rejection, leading to a failure of the rat’s own blood vessels to achieve vascular remodeling with the lumen formed by HUVECs. (ii) The rat’s own blood vessels grew from the outside of the implant to the inside, resulting in fewer internal blood vessels inside than outside. Further research is needed to confirm our hypothesis. Although the effect of subcutaneous vascular remodeling in rats is not good, it is preliminarily proven that millimeter-sized implants can achieve vascular remodeling *in vivo*.

In addition, fibrin was found to improve cell survival and induce neovascularization *in vivo* [51, 52]. Unfortunately, our implants (experimental group and control group) both contained fibrin, and thus, we cannot determine whether the vascular remodeling of subcutaneous implants in rats is predominantly mediated by fibrin or vascularized engineered myocardial tissue. However, the number of blood vessels in the inner ring of the experimental group implant was significantly lower than that in the outer ring. If fibrin plays a major role in vascular remodeling, the number of vessels in the inner- and outer-rings might not be different. Therefore, we reasonably speculate that the vascularized engineered myocardial tissue plays a leading role in the subcutaneous vascular remodeling in rats.

We also constructed several batches of vascularized engineered myocardial tissues and carried out static culture for up to 4 weeks to evaluate the differences among different batches of tissue. It is difficult to maintain consistency in the structure and function of a vascularized engineered myocardial tissue formed through the self-organization of seed cells from different batches of tissue formation (early or late stages), in terms of the size and number of lumens, the frequency and amplitude of spontaneous pulsation, and the rate of tissue collapse, which varied among the different batches. Undoubtedly, 3D printing technology has advantages for precise control and maintaining consistency [53]. This method of cell self-organization cannot be used to arrange hiPSC-CMs in biomaterials into a layered structure, such as the natural myocardium, as with 3D printing and other technologies. This could be an important factor that ultimately affects the formation of a consistent pulsation rate and amplitude in the engineered myocardium, as well as the orientation of blood vessels in the tissue [23].

Although there is still a large gap between the thickness of the vascularized engineered myocardial tissue developed by our group and that of the normal left ventricular wall [48], the proposed method is simpler than layer-by-layer or 3D printing. In particular, the tissue and its lumen-like structures are not supported by high-hardness materials, and they still have lasting mechanical properties. These conclusions provide preliminary evidence suggesting that the constructed millimeter-scale vascularized engineered myocardium has the potential for transplantation *in vivo*, but further animal experiments are needed to evaluate its potential for clinical use.

## Conclusion

In summary, we successfully constructed vascularized engineered myocardial tissue with a millimeter-sized spherical structure via hiPSC-CMs and HUVECs self-organization in a mixed gel with fibrin and collagen І, using a three-stage culture method. This vascularized engineered myocardium had autonomous beats that were close to those of the normal human heart, and the microvascular diameter formed by HUVECs inside exceeded 100 μm. In addition, these tissues also achieved surface re-vascularization *in vitro* and vascular remodeling *in vivo*. This method for constructing vascularized engineered myocardial tissue does not require high-end manufacturing equipment or cutting-edge preparation techniques, and the constructed tissue has potential clinical value, subject to preliminary evaluation.

## Supplementary Material

rbad117_Supplementary_Data
