# Supplementary material for: Construction of millimeter-scale vascularized engineered myocardial tissue using a mixed gel
Source: Regen Biomater. 2023 Dec 29;11:rbad117. doi: 10.1093/rb/rbad117 (PMC10786677; doi:10.1093/rb/rbad117)
Supplement: rbad117_Supplementary_Data [file rbad117_supplementary_data.zip › Supplementary Information file(FINAL version).docx]

**Methods and results that need special explanation Section 1-6**

**Section 1. Fibrin gel component ratio exploration experiment**

Two kinds of mixed gels containing different concentrations of fibrin (2.5 mg/mL and 5 mg/mL) were prepared, and then mixed HUVEC (5 × 10^6^ cell/mL) in the gel for static experiment of 3D tube forming ability. The microscope image of the experimental process is shown in Figure S1. On the first day, the edge of the mixed gel containing 2.5 mg/mL fibrin curled and fell off the climbing piece (indicated by the red arrow in Figure S1 A), and there were gaps in the gel (indicated by the red asterisk in Figure S1 A). Some HUVECs grew on the climbing piece. Although the edge of the mixed gel containing 5 mg/mL fibrin was slightly shrunk, the gel did not peel off from the climbing piece (indicated by the red triangle in Figure S1 B). On the third day of culture, the mixed gel containing 2.5 mg/mL fibrin further curled and the gap in the gel increased (indicated by the red asterisk in Figure S1 A), and a large number of HUVECs grew on the climbing piece. Although the mixed gel containing 5 mg/mL fibrin further shrinks, the cells still grow in 3D in the gel, and the cells at the edge of the gel sprout outward significantly (indicated by the red triangle in Figure S1 B). The results showed that the 3D growth state of HUVECs in the mixed gel containing 5 mg/mL fibrin was more stable and easier to sprout than that in the mixed gel containing 2.5 mg/mL fibrin. However, we did not carry out pre-experiments on the mixed gels containing different fibrins suitable for hiPSC-CMs 3D growth. The is because the myocardial tissue we construct needs to achieve two main purposes in stages. In the first culture stage (0–24h), HUVECs could rapidly and spontaneously form tubes in the mixed gel. In the second culture stage (Day 2–5), hiPSC-CMs could beat spontaneously in the mixed gel. So when we determined that the mixed gel containing 5 mg/mL fibrin was more suitable for rapid tube formation of HUVEC, we used the mixed gel of this component to conduct 3D static culture of hiPSC-CM. As shown in Figure 2G in the manuscript, hiPSC-CMs could grow in 3D in the mixed gel containing 5 mg/mL fibrin and showed obvious spontaneous pulsation on the third day.

**
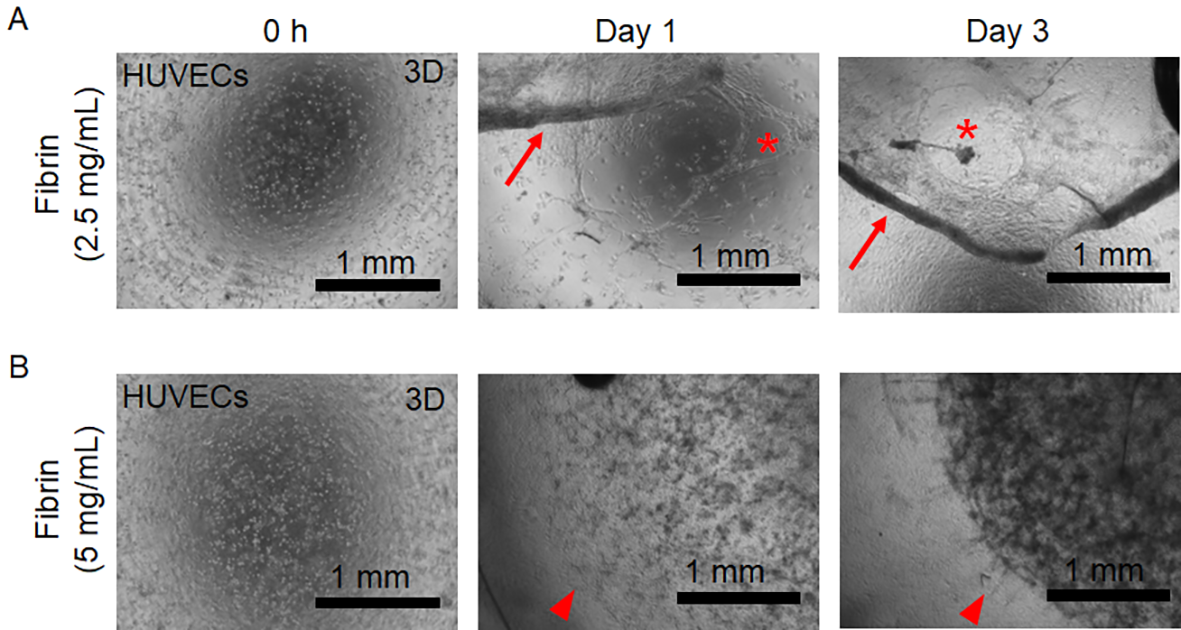
**

Figure S1. The 3D tube formation of HUVECs in the mixed gel containing two different concentrations of fibrin. (A) The content of fibrin is 2.5 mg/mL. (B) The content of fibrin is 5 mg/mL.

**Section 2. Viscoelastic testing of Fibrin gel**

The viscoelasticities of the fibrinogen solution and fibrin gel were measured using a hybrid rheometer (TA Instruments, USA). Bovine fibrinogen solution (5 mg/mL) and bovine fibrinogen solution (5 mg/mL) containing thrombin (3 U/mL) were placed on the hybrid table of the hybrid rheometer for 5 min, and the viscoelasticity of the samples was tested. The test parameters of the hybrid rheometer: Temperature of the hybrid table, 37 °C; Temperature of test, 37 °C; Rate of shear, 0.001–1000 (1/S); Sampling points, 31; 3 samples/type; and 1 time/sample. The viscosity and stress of the samples as a function of the shear rate were recorded using software from the hybrid rheometer, and plots were drawn.

The results of viscoelastic testing (Figure S2) showed that the viscosities of fibrin before and after cross-linking are significantly different. When the shear rate is between 10-3 and 10-1 (1/S), the viscosity of the fibrinogen solution decreased with an increase in shear force. The viscosity of the fibrin gel increased with an increase in shear force. Under low stress, the fibrin gel behaves as a rigid body. However, it serves as a viscous fluid under high stress, and its fluidity is linear, as indicated by the red dotted line in figure S1. This phenomenon was similar to the mechanical properties of a Bingham body. This mechanical property is generally observed in colloids. The stress curves show that the stress of the fibrin gel was significantly higher than that of the fibrinogen solution. However, when the shear rate was between 10^-1^ and 10^1^ (1/S), the viscosities of the two samples decreased sharply with increasing shear rates. This is because the separation of hydrated fibrin from the fibrin gel at a high shear rate during testing.

The viscoelasticity test results showed that the viscoelasticity changed significantly before and after cross-linking, and fibrinogen formed a scaffold material with hardness and elasticity under the action of thrombin. The viscosity of fibrin gel (5mg/mL) is within 1kP, which was suitable for the growth and harvest of seed cells. Therefore, when there is no spontaneous pulsation at the early period of micro-tissue formation, the low hardness of the fibrin gel was suitable for HUVECs to bud and form lumen-like structures. When the micro-tissue is spontaneously pulsating, the fluid properties of fibrin gel could adapt to the spontaneous pulsation of hiPSC-CMs. Therefore, in different mechanical environments, fibrin gel with solid and fluid properties could simultaneously meet the needs of seed cells for different growth stress environments.


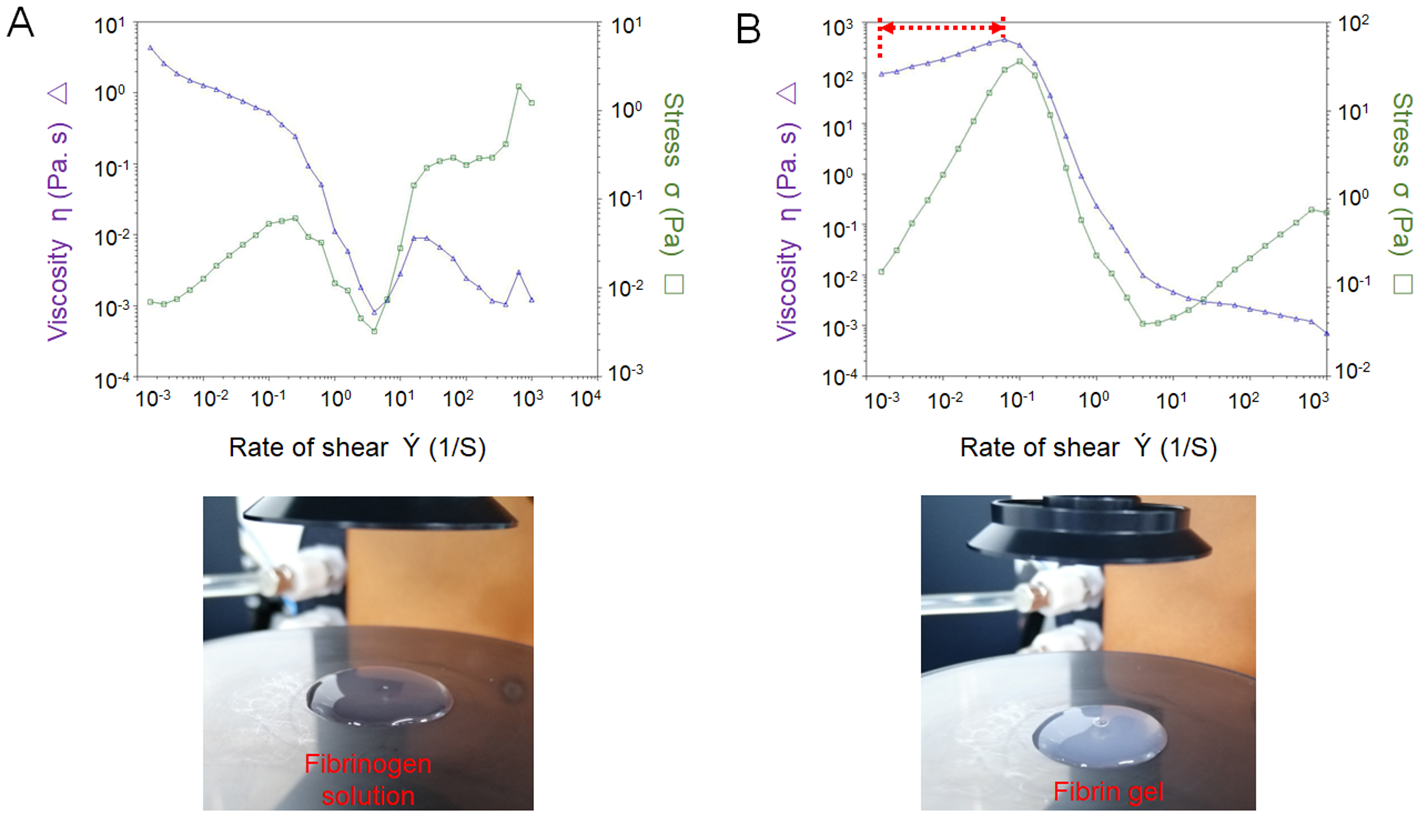


Figure S2. Results of viscoelastic testing of fibrinogen solution and fibrin gel. (A) Viscoelastic test plot of the fibrinogen solution and photographs of the tested sample. (B) Viscoelastic test plot of the fibrin gel and photographs of the tested sample.

**Section 3. Characterization of hiPSC-CMs and HUVECs**

hiPSC-CMs were characterized by evaluating the expression of cTnT and F-actin markers using IF staining. The specific markers cTnT and F-actin presented a sarcomere-like morphology. HUVECs were characterized by measuring the expression of vWF and F-actin markers using IF staining. The cells grew in two-dimensional spreading and were positive for the specific marker vWF.


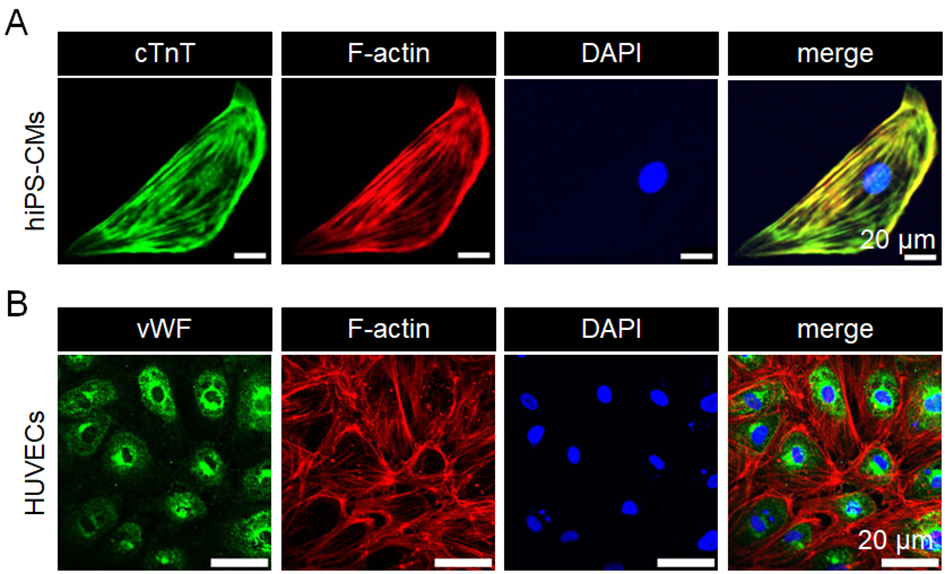


Figure S3. Cytological characterization results of hiPSC-CM and primary HUVEC. (A) Fluorescence microscope pictures of IF staining of hiPSC-CMs. (B) Fluorescence microscope pictures of IF staining of primary HUVECs.

**Section 4. Constructing non-vascularized engineered myocardial tissue**

The materials and methods for constructing non-vascularized engineered myocardial tissue was the same as those for constructing vascularized engineered myocardial tissue, except that the HUVECs suspension was replaced with a cardiomyocyte support medium. In addition, the mixed medium used for static culture was replaced with the cardiomyocyte maintenance medium.

**Section 5. Design and Fabrication of Single Channel PDMS Micro-Chip**

A single channel PDMS micro-chip is designed with Auto CAD software (Autodesk, USA) according to the experimental requirements. Preparation process of single channel PDMS micro-chip (Figure S4): The cleaned and dried tungsten alloy (L×W×H = 1.5×0.6×0.1 cm) is used as the structural male mold and fixed at the bottom of the glass culture dish. Prepare polydimethylsiloxane prepolymer (PDMS, Sylgard 184, Dow Corning, USA) solution (V: V = 10:1). Cover the PDMS prepolymer solution with no air bubble on the male mold structure, place it in an oven at 60 ℃ for curing for 4 hours, and then cut the cured PDMS female mold with a scalpel. At both ends of the female mold structure, a punch is used to punch an inlet and an outlet respectively to form an inlet and an outlet, and then the perforated female mold and the glass slide are reversely packaged by hot pressing to form a single channel PDMS micro-chip. The micro-chip is sterilized at high temperature and dried before use.


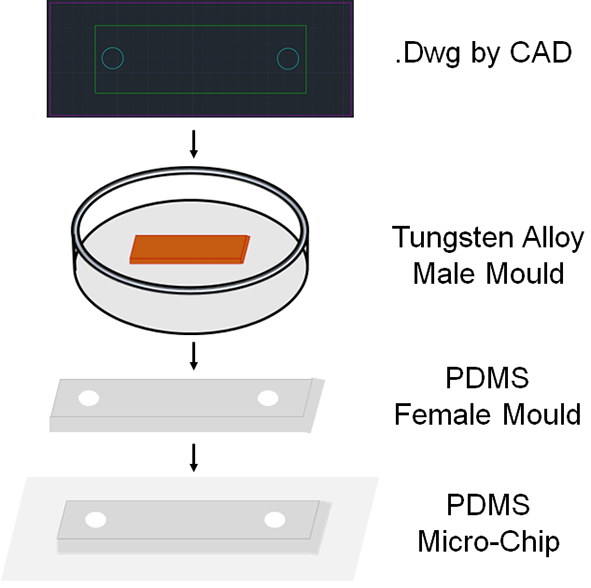


Figure S4. Schematic diagram of the preparation process of a single channel PDMS micro-chip.

**Section 6. Verified the feasibility of the experimental scheme of HUVECs wrapping the tissue *in vitro*.**

This experiment aimed to verify whether HUVECs could wrap tissue for growth. 250 μL HUVECs suspensions (2×10^7^ cells/mL), 100 μL bovine fibrinogen solution, 20 μL collagen I, 30 μL thrombin solution, and 600 μL mixed medium were thoroughly mixed on ice to form a pre-polymerized solution. A non-vascularized engineered myocardial tissue was injected into the entrance of the micro-chip (tissue without HUVECs would not cause interference with the growth of HUVECs around the tissue, which is convenient for observing the experimental results). The pre-polymerized solution containing HUVECs was injected into the micro-chip to make the HUVECs wrapped around the tissue. It was subsequently placed in an incubator at 37 ℃ and 5% CO_2_ for 10 min. The mixed culture medium was then injected into the micro-chip, and the mixed culture was added simultaneously until the inlet and outlet of both micro-chips were completely covered. Next, it was transferred to the incubator at 37 ℃ and 5% CO_2_ for static culture. After 24 h, the gel with HUVECs in the micro-chip, except the target culture, were washed away with the mixed medium. When HUVECs wrapped the tissue and grew for 5 days, IF staining was performed.

The tissue sections were scanned using laser confocal scanning, and 3D reconstruction was performed (Figure S5). The results showed that HUVECs wrapped the tissue and grew.


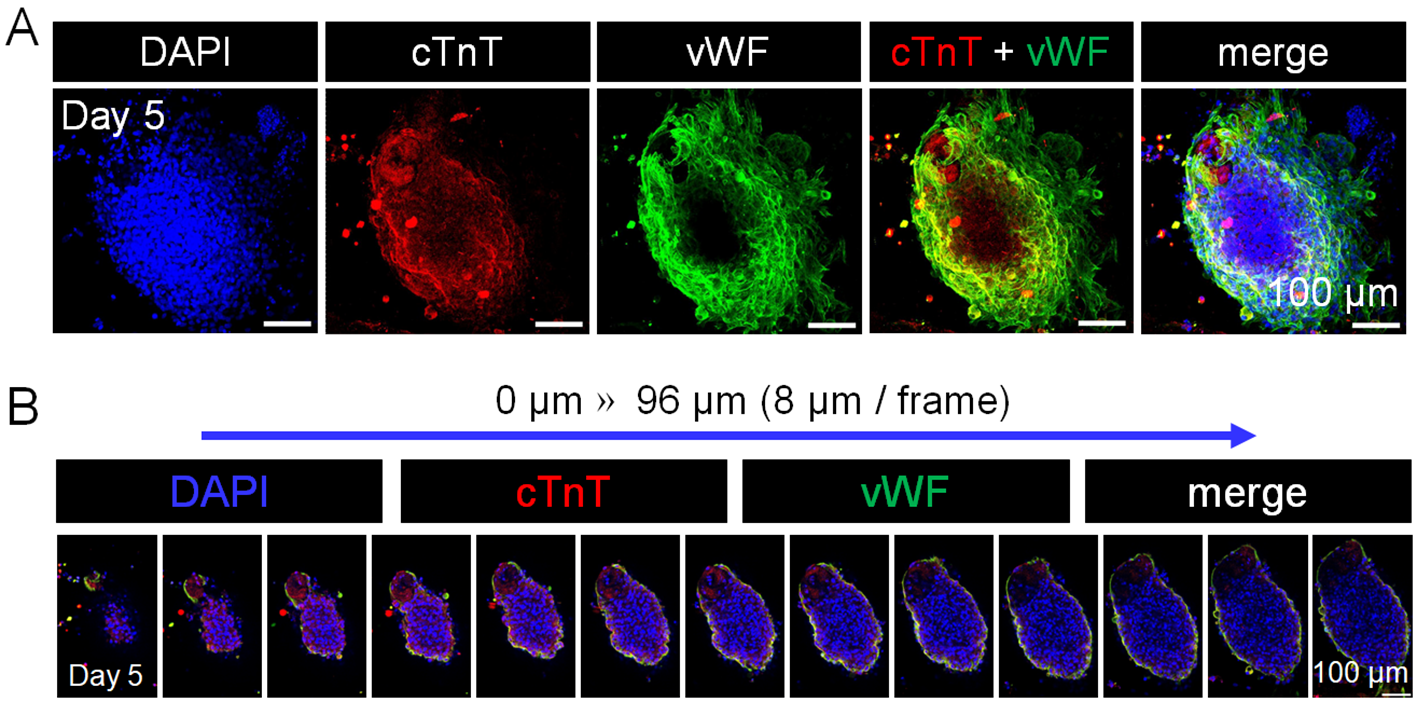


Figure S5. IF staining images of HUVECs wrapping myocardial tissue growing to day 5. (A) 3D images reconstructed by fluorescence confocal scanning. (B) Cross-section IF staining images taken by fluorescence confocal microscope tomography.

**Captions for movies S1-S4**

**Movie S1**: The process video of its spontaneous rhythmic pulsation was captured under a microscope of the vascularized engineered myocardial tissue cultured in a static state for 2–14 days. Its spontaneous pulsation process gradually became faster and then slower.

**Movie S2**: The video of vascularized engineered myocardial tissue with a spontaneous pulsation rate of nearly 70 beats per minute was captured under a microscope in static culture.

**Movie S3**: The video under the microscope showed that the spontaneous rhythmic pulsation of the vascularized engineered myocardial tissue cultured statically for 35 days was weak.

**Movie S4**: The spontaneous rhythmic pulsation video of the vascularized engineered myocardial tissue wrapped in fibrin film and cultured for 15 days was captured under a microscope.
